# WHAT FACTORS ARE ASSOCIATED WITH CRITICAL MEDIA LITERACY IN LATER LIFE?

**DOI:** 10.1093/geroni/igad104.2213

**Published:** 2023-12-21

**Authors:** Hayoung Park, Bomi Choi, Si Young Song, Miseon Kang

**Affiliations:** Yonsei University, Seoul, Republic of Korea; Yonsei University, Seoul, Republic of Korea; Kyung Hee University, Seoul, Republic of Korea; Yonsei University, Seoul, Republic of Korea

## Abstract

Who consumes new information from the media more critically? This study sought to empirically explore relevant factors associated with critical media literacy in later life. For the analyses, the 2021 Korean Media Panel Survey was utilized and the sample included 2,352 older Korean adults aged 65 and above (M=75.48, SD=7.34, range=65-106). Critical media literacy was measured by the mean of ten questions asking how critically evaluate the facticity, validity, intentions, and usefulness of new information from the media. Possible relevant factors include age, gender, education level, employment status, marital status, income level, and the ability to use digital technology (PC and smartphone). The results from the linear regression analyses showed that age, income level, and the ability to use digital technology were significantly associated with critical media literacy in later life. Older adults who are younger and have higher levels of income and the ability to use digital technology tend to evaluate new information from the media more critically. The findings from this study imply the importance of specialized media literacy education as social policy for older adults, especially those who are older and have lower levels of income and the ability to use digital technology. This study is significant as it empirically examined various factors related to critical media literacy in later life to inform intervention efforts to reduce misperceptions induced by misinformation that is rampant in this “information era”. Future studies are needed to investigate significant factors associated with critical media literacy in later life via various contexts.

